# Association of myosteatosis with treatment response and survival in patients with hepatocellular carcinoma undergoing chemoembolization: a retrospective cohort study

**DOI:** 10.1038/s41598-023-31184-9

**Published:** 2023-03-09

**Authors:** Kittipitch Bannangkoon, Keerati Hongsakul, Teeravut Tubtawee, Natee Ina, Ply Chichareon

**Affiliations:** 1 Department of Radiology, Faculty of Medicine, Prince of Songkla University, Hat Yai, Songkhla Thailand; 2 Cardiology Unit, Division of Internal Medicine, Faculty of Medicine, Prince of Songkla University, Songkhla, Thailand; 3 Naradhiwas Rajanagarindra Heart Center, Songklanagarind Hospital, Songkhla, Thailand

**Keywords:** Cancer therapy, Liver cancer

## Abstract

Patients with hepatocellular carcinoma (HCC) have poor prognosis and have frequent treatment-related toxicities resulting in cancer-associated cachexia. This study aimed to determine the association of myosteatosis and sarcopenia on mortality in patients with HCC treated with transarterial chemoembolization (TACE). Six hundred and eleven patients diagnosed with HCC and underwent TACE at a tertiary care center between 2008 and 2019 were included. Body composition was assessed using axial CT slices at level L3 to calculate the skeletal muscle density for myosteatosis and skeletal muscle index for sarcopenia. The primary outcome was overall survival while the secondary outcome was TACE response. Patients with myosteatosis had a poorer TACE response than patients without myosteatosis (56.12% vs. 68.72%, adjusted odds ratio [OR] 0.49, 95% confidence interval [CI] 0.34–0.72). The rate of TACE response in patients with sarcopenia was not different from those without sarcopenia (60.91% vs. 65.22%, adjusted OR 0.79, 95% CI 0.55–1.13). Patients with myosteatosis had shorter overall survival than without myosteatosis (15.9 vs. 27.1 months, *P* < 0.001). In the multivariable Cox regression analysis, patients with myosteatosis or sarcopenia had higher risk of all-cause mortality than their counterparts (adjusted hazard ratio [HR] for myosteatosis versus no myosteatosis 1.66, 95% CI 1.37–2.01, adjusted HR for sarcopenia versus no sarcopenia 1.26, 95% CI 1.04–1.52). Patients with both myosteatosis and sarcopenia had the highest 7 year mortality rate at 94.45%, while patients with neither condition had the lowest mortality rate at 83.31%. The presence of myosteatosis was significantly associated with poor TACE response and reduced survival. Identifying patients with myosteatosis prior to TACE could allow for early interventions to preserve muscle quality and might improve prognosis in HCC patients.

## Introduction

Hepatocellular carcinoma (HCC) is an aggressive type of malignancy that is the third leading cause of cancer-related mortality worldwide^1^. Transarterial chemoembolization (TACE) displays a good response rate and clinical benefit for patients with inoperable HCC^2,3^. In some patients, TACE enables the conversion of unresectable and locally advanced liver cancer to operable cancer leading to an increase in survival outcome^4^. While TACE is a commonly used treatment for HCC, many patients do not respond well to it and have poor survival outcomes^5^. This is important for patients who receive chemoembolization because, although chemotherapy can give survival benefits to patients, it also causes liver toxicity and can lead to physical inactivity.

Skeletal muscle density (SMD) is known as a poor prognostic factor in patients with malignancies and is highly associated with tumor progression and mortality^6–8^. In principle, fat infiltration in muscle tissue appears as a lower density on CT scans compared to regular muscle tissue measured in Hounsfield units (HU)^9^. Myosteatosis (low SMD) indicates intramuscular fat deposition and low-grade skeletal muscle, which is correlated to poor muscle strength^10^.

Studies on body compositions in patients with HCC undergoing TACE are lacking and frequently focus on sarcopenia, not myosteatosis^11–13^. To the best of our knowledge, the association between myosteatosis and response to chemoembolization in HCC patients has not been well established. Since an abdominal CT scan is part of the routine pre-and postoperative evaluation for patients with liver cancer^14^, assessment of skeletal muscle could be employed in clinical practice. In this study, we aimed to determine the association of myosteatosis with TACE response and survival outcome in patients with HCC. The results can be used to provide an early screening modality in HCC patients to identify patients at risk of not responding well to TACE. Furthermore, early preventive strategies may potentially improve the outcome for patients with myosteatosis.

## Patients and methods

### Ethical approval

This study adhered to the standards of the Declaration of Helsinki and current ethical guidelines. Ethical approval was obtained by the institutional review board of the Faculty of Medicine, Prince of Songkla University and Songklanagarind Hospital (REC.65-317-7-1). The requirement for informed consent for this study was waived by the Institutional Review Board of the Faculty of Medicine, Prince of Songkla University and Songklanagarind Hospital as the study was a retrospective study.

### Patient population

Patients diagnosed with HCC and underwent TACE from January 2008 to December 2019 were included and analyzed. The inclusion criteria were as follows: (1) age greater than 18 years; (2) HCC diagnosis by imaging or histological findings according to the American Association for the Study of Liver Disease guidelines^15^; (3) initial treatment with conventional TACE; (4) HCC with Barcelona Clinic Liver Cancer (BCLC) stage A, B, or C (subsegmental or segmental portal vein tumor thrombosis); (5) available medical records; and (6) Child–Pugh class A or B. The exclusion criteria were as follows: (1) absence of imaging data; (2) inability to measure the skeletal muscle mass; (3) concomitant malignancies; and (4) history of HCC rupture.

### TACE protocol and treatment schedule

All patients with HCC were treated using conventional TACE by two experienced interventional radiologists who had at least 10 years of experience. We administered a mixture of iodized oil (range: 4–16 mL) and doxorubicin hydrochloride (range: 5–50 mg) or mitomycin-C (range: 10–20 mg) via the tumor-feeding hepatic arteries. We finished the procedure when the tumor feeding branch was completely embolized by gelatin sponge particles. The decision to repeat TACE session was made on demand at an interval of 6–12 weeks in patients with favorable liver function and performance status.

We evaluated baseline CT scans before TACE and 1-month post-TACE to evaluate TACE responses. The treatment response was assessed based on the imaging studies of the patients, which were either 4-phase contrast-enhanced CT scan or dynamic magnetic resonance imaging within 1 month after the initial TACE. The modified Response Evaluation Criteria in Solid Tumors (mRECIST) was used to assess radiological changes of HCC after treatment^16^. The criteria have four categories; complete response (CR); partial response (PR); stable disease (SD); and progressive disease (PD). Complete or partial response in the imaging study at 1-month post-TACE was classified as TACE response whereas stable or progressive disease was defined as no response. Assessment of tumor response was reviewed independently by two radiologists with expertise in liver imaging to minimize variability. In cases of disagreement, the final decision was obtained by consensus.

### Measurement and definition of body composition

CT scans within 1 month prior to TACE or in the first post-TACE were selected to measure body composition. Pre-TACE scans were preferentially chosen. When these were unavailable, the earliest post-TACE scans were used in the study. The CT images at the level of the third lumbar vertebra (L3) were carefully chosen and archived as Digital Imaging and Communications in Medicine (DICOM) data. All DICOM data calculated body composition using in-house software developed by MATLAB (The MathWorks, Natick, MA, USA) and freeware Python 3.6.13 (Anaconda, Inc.), to generate the measurement model based on neural network architecture also known as UNet. The valid accuracy of the model was 99.17% and validity of the intersect over union co-efficiency was 89.40%^17^.

The L3 skeletal muscle index (SMI) is used to identify sarcopenia and is calculated by dividing the cross-sectional area of the muscle by the square of the patient's height (cm^2^/m^2^). Sarcopenia was defined as SMI ≤ 36.2 cm^2^/m^2^ and ≤ 29.6 cm^2^/m^2^ for males and females, respectively^11^. The areas of the abdominal wall and back muscles were used to calculate the SMD based on the areas of the pixels with attenuation between − 29 and + 150 HU. Myosteatosis was defined as SMD ≤ 44.4 HU or ≤ 39.3 HU in males and females, respectively^11^. In addition, patients were classified into four groups according to their sarcopenia and myosteatosis status (Group A—neither sarcopenia nor myosteatosis, Group B—sarcopenia without myosteatosis, Group C—myosteatosis without sarcopenia, and Group D—sarcopenia with myosteatosis).

### Data collection

The following data were collected: demographic information (age, sex, body mass index; clinical history (hepatitis B or C virus carriers, alcohol consumption, diabetes, hypertension, cardiovascular disease, pulmonary disease, chronic kidney disease, Child–Pugh class, and BCLC staging); laboratory data (levels of aspartate transaminase, alanine transaminase, total bilirubin, albumin, platelet count, and serum alpha-fetoprotein); tumor factors (size and number of tumors); and imaging response within 1 month after the initial TACE. The up-to-seven criteria were calculated by the summation of the largest tumor diameter in cm and the number of tumors. Two strata of the scores included the summation of scores ≤ 7 or > 7^18^. The clinical and laboratory information were collected from the electronic medical records.

### Statistical analysis

Data analyses were performed using R software, version 4.2.0 (R foundation, Vienna, Austria). Baseline characteristics are shown as mean ± SD for normally distributed continuous variables or median (interquartile range) for those with a skewed distribution. Discrete variables are shown as counts (percentages). Differences between groups were evaluated using t-test or continuous variables and Pearson’s chi-square test or Fisher’s exact test for categorical data.

The cumulative overall survival (OS) rate was estimated using the Kaplan–Meier method and significant differences in survival distributions were tested by the log-rank test. Vital status (death or alive) was obtained from the civil registry up to December 31, 2021. Survival time was defined as the interval between the first TACE session for HCC and death or December 31, 2021 if the patient was alive.

A scatter plot was used to show the correlation between SMD and SMI. The degree of correlation was quantified with the correlation coefficient (R^2^). The association between myosteatosis or sarcopenia and mortality was assessed in multivariable Cox regression models. Adjusted variables included age, chronic lung disease, and chronic kidney disease since these variables had prognostic impact on mortality and were associated with myosteatosis and sarcopenia^19–23^. Multivariable logistic regression analysis was used to determine the association between myosteatosis or sarcopenia and TACE response (response versus no response). The adjusted variables in the logistic regression model were the variables used in the adjusted Cox model. *P* values less than 0.05 were considered to be statistically significant.

## Results

### Patient and tumor characteristics

**Figure 1:**
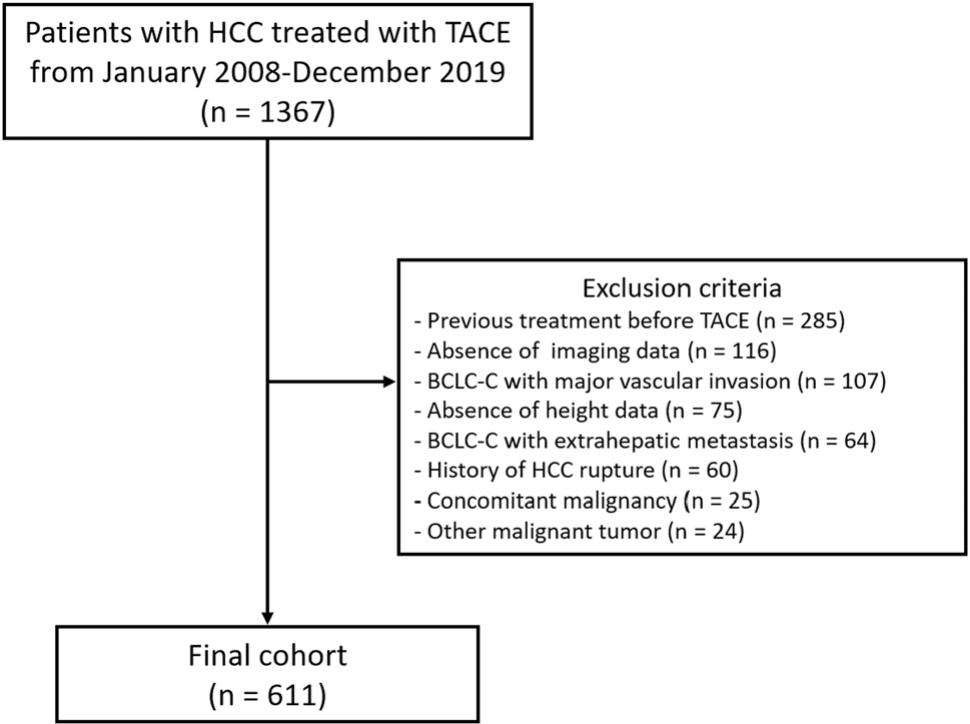
Flow of study patients.

A total of 611 patients with HCC were included in this study (Fig. 1). The patients and tumor characteristics are shown in Table 1. The mean patient age was 61.4 ± 10.9 years and males were predominant in this cohort (72.8%). Half of the patients (50.0%) had normal weight. Hepatitis B virus infection was the most common cause of liver cirrhosis (49.3%). Diabetes (27.7%) and hypertension (27.2%) were the two most common comorbidities. The BCLC stage distribution was as follows: stage A, 172 (28.0%); stage B, 406 (66.4%); and stage C, 33 (5.4%).

**Table 1:** Baseline characteristics of the study participants who underwent TACE.

Characteristics	Value
Patients, N	611
Age, mean ± SD	61.43 ± 10.90
Gender male, N (%)	445 (72.8)
Body mass index (kg/m^2^)	
< 20.0 (underweight), N (%)	106 (17.3)
20.0–24.9 (normal weight), N (%)	305 (50.0)
≥ 25.0 (overweight/obese), N (%)	200 (32.7)
Etiology, N (%)	
HBV/HCV/HBV + HCV/Alcohol/others	301 (49.3)/135 (22.1)/7 (1.1)/65 (10.6)/103 (16.9)
Comorbidity, N (%)	
Diabetes	169 (27.7)
Hypertension	166 (27.2)
Cardiovascular disease	39 (6.4)
Pulmonary disease	35 (5.7)
Chronic kidney disease	26 (4.3)
Child–Pugh class, N (%)	
A/B	465 (76.1)/146 (23.9)
AST (IU/L), median (IQR)	61.0 (42.0,88.0)
ALT (IU/L), median (IQR)	38.0 (25.0,61.0)
Total bilirubin (mg/dL), median (IQR)	0.81 (0.53,1.32)
Albumin (g/dL), mean ± SD	3.53 ± 0.54
Platelet count (× 10^3^/mm^3^), median (IQR)	116 (74,189)
BCLC-staging, N (%)	
A/B/C	172 (28.2)/406 (66.4)/33 (5.4)
Alpha-fetoprotein (ng/mL), N (%)	
< 200, ≥ 200	418 (68.4)/193 (31.6)
Tumor size (cm), N (%)	
≤ 3, 3–5, > 5	171 (28.0)/173 (28.3)/267 (43.7)
Number of tumors, N (%)	
1, 2–3, > 3	257 (42.0)/188 (30.8)/166 (27.2)
TACE sessions, median (IQR)	2 (1–4)
SMI (cm^2^/m^2^), median (IQR)	
Male	39.9 (34.8,44.6)
Female	30.5 (27.0,34.3)
SMD (HU), median (IQR)	
Male	46.0 (41.9,50.2)
Female	39.7 (35.0,43.3)

*SD* standard deviation, *IQR* interquartile range, *HBV* hepatitis B virus, *HCV* hepatitis C virus, *AST* aspartate transaminase, *ALT* alanine transaminase, *BCLC* Barcelona Clinic Liver Cancer, *TACE* transarterial chemoembolization, *SMI* skeletal muscle index, *SMD* skeletal muscle density.

### Correlation between SMI and SMD

**Figure 2:**
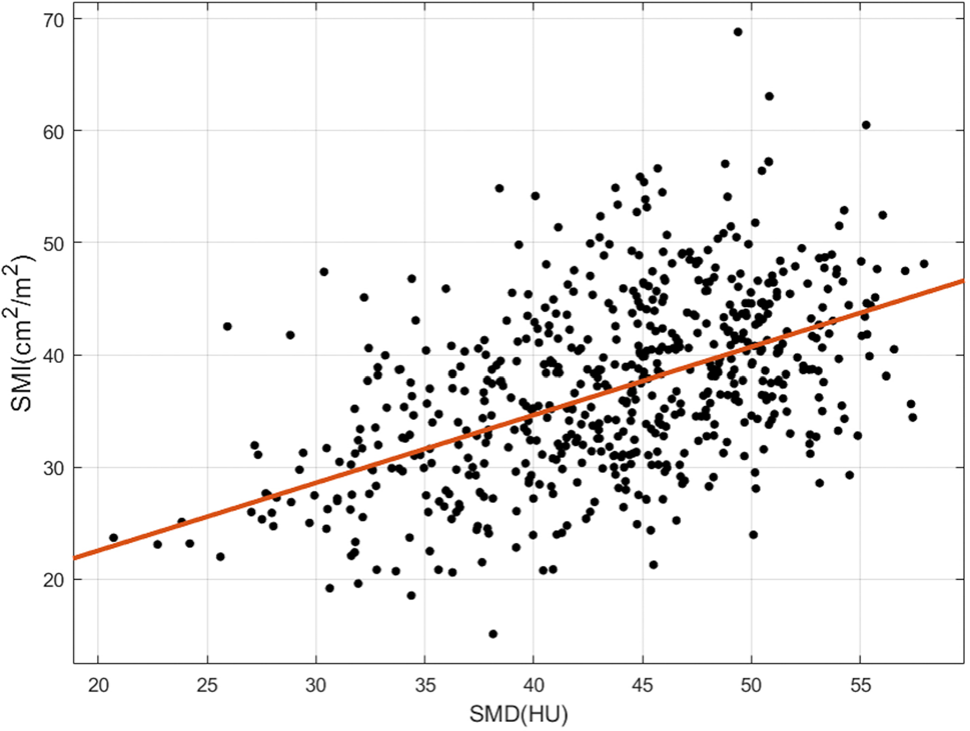
Correlation between skeletal muscle index (SMI) and skeletal muscle density (SMD).

The prevalences of low SMI and low SMD were 197 (32.2%) and 237 (38.8%) patients. The correlation between SMI and SMD is shown in the scatter plot (Fig. 2). A positive correlation between SMI and SMD was demonstrated (*R*^2^ = 0.238, *P* < 0.001).

### Associations between sarcopenia and myosteatosis and TACE response and complications

Treatment response and complications after TACE were evaluated according to sarcopenia and myosteatosis status (Table 2). Among the 611 patients, 390 responded well to TACE while 221 had a poor response. Patients with myosteatosis had a lower rate of TACE response than patients without myosteatosis (56.1% vs. 68.7%, adjusted odds ratio [OR] 0.49, 95% confidence interval [CI] 0.34–0.72). The rate of TACE response in the patients with sarcopenia was not different from those without sarcopenia (60.9% vs. 65.2%, adjusted OR 0.79, 95% CI 0.55–1.13). Postembolization syndrome and liver decompensation were numerically, although not statistically, higher in the myosteatosis group compared to patients without myosteatosis (21.5% vs. 19.3%, *P* = 0.564 and 5.5% vs. 4.0%, *P* = 0.515). Patients in group B had a similar chance for TACE response to those in group A (adjusted OR 0.73, 95% CI 0.43–1.24). Compared with group A, the chance for TACE response was significantly lower in group C (adjusted OR 0.42, 95% CI 0.27–0.67) and group D (adjusted OR 0.50, 95% CI 0.31–0.81).

**Table 2:** TACE response and complication according to myosteatosis or sarcopenia.

Parameter	No sarcopenia (N = 414)	Sarcopenia (N = 197)	*P* value	No myosteatosis (N = 374)	Myosteatosis (N = 237)	*P* value
TACE response	120 (60.9)	270 (65.2)	0.345	133 (56.1)	257 (68.7)	0.002
Postembolization syndrome	76 (18.4)	47 (23.9)	0.140	72 (19.3)	51 (21.5)	0.564
Liver decompensation	17 (4.1)	11 (5.6)	0.542	15 (4.0)	13 (5.5)	0.515

*TACE* transarterial chemoembolization.

### Association between sarcopenia or myosteatosis and survival

The median OS time for the cohort was 22.1 months (95% CI 18.7–25.1 months). The 1-, 3-, and 5-year OS rates were 67.8%, 32.5%, and 19.0%, respectively. Patients with myosteatosis also had a lower OS time than patients without myosteatosis (15.9 vs. 27.1 months, *P* < 0.001). Patients with sarcopenia had a lower OS time than patients without sarcopenia (16.6 vs. 23.8 months, *P* = 0.011).

**Figure 3:**
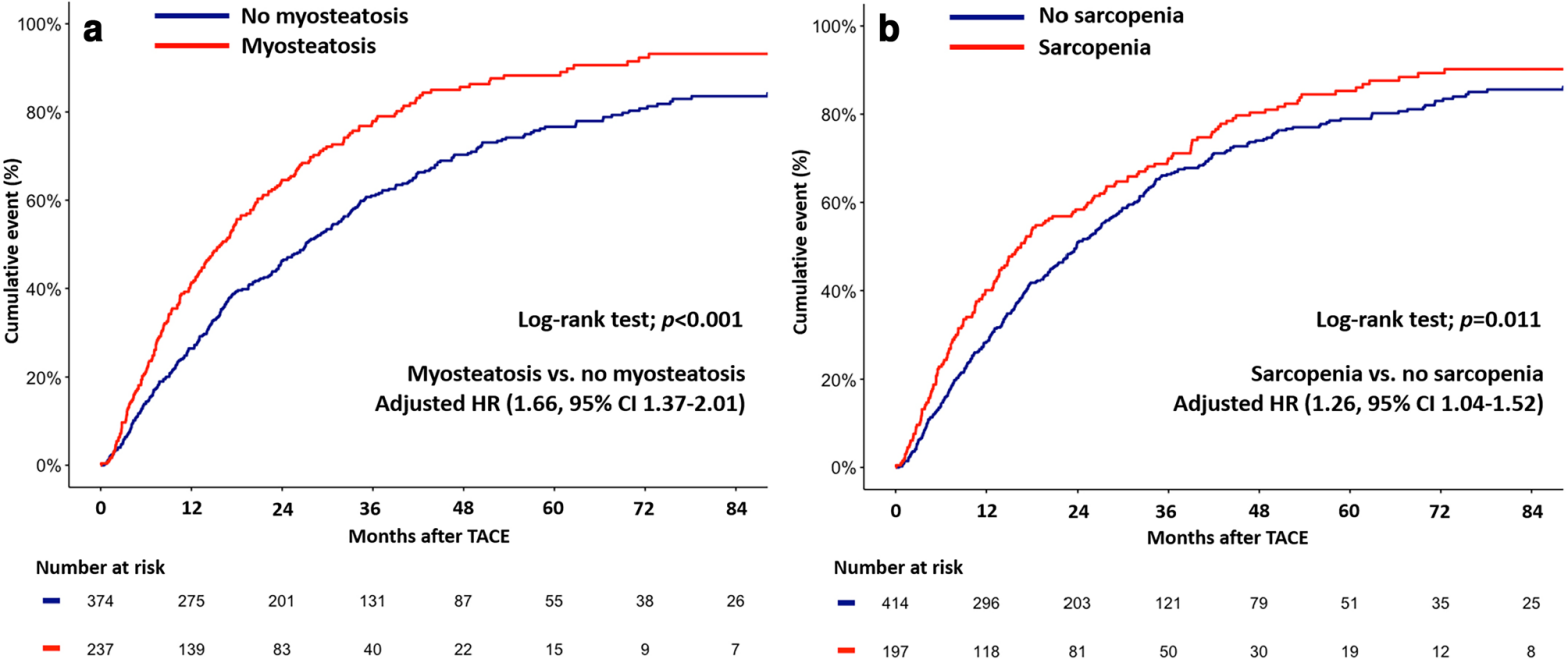
All-cause mortality of patients (**A**) with or without myosteatosis and (**B**) with or without sarcopenia. *adjusted by age, chronic lung disease, and chronic kidney disease.

The overall all-cause mortality rate at seven years following TACE was 87.13%. Patients with myosteatosis had a higher rate of mortality than patients without myosteatosis (93.18% vs. 83.58%, *P* < 0.0001) (Fig. 3). The rates of 7-year all-cause mortality were 90.2% and 85.59% in patients with and without sarcopenia, respectively (*P* = 0.011). In the multivariable Cox regression analysis, patients with myosteatosis or sarcopenia had a higher risk of all-cause mortality than their counterparts (adjusted hazard ratio [HR] for myosteatosis versus no myosteatosis 1.66, 95% CI 1.37–2.01, adjusted HR for sarcopenia versus no sarcopenia 1.26, 95% CI 1.04–1.52).

**Figure 4:**
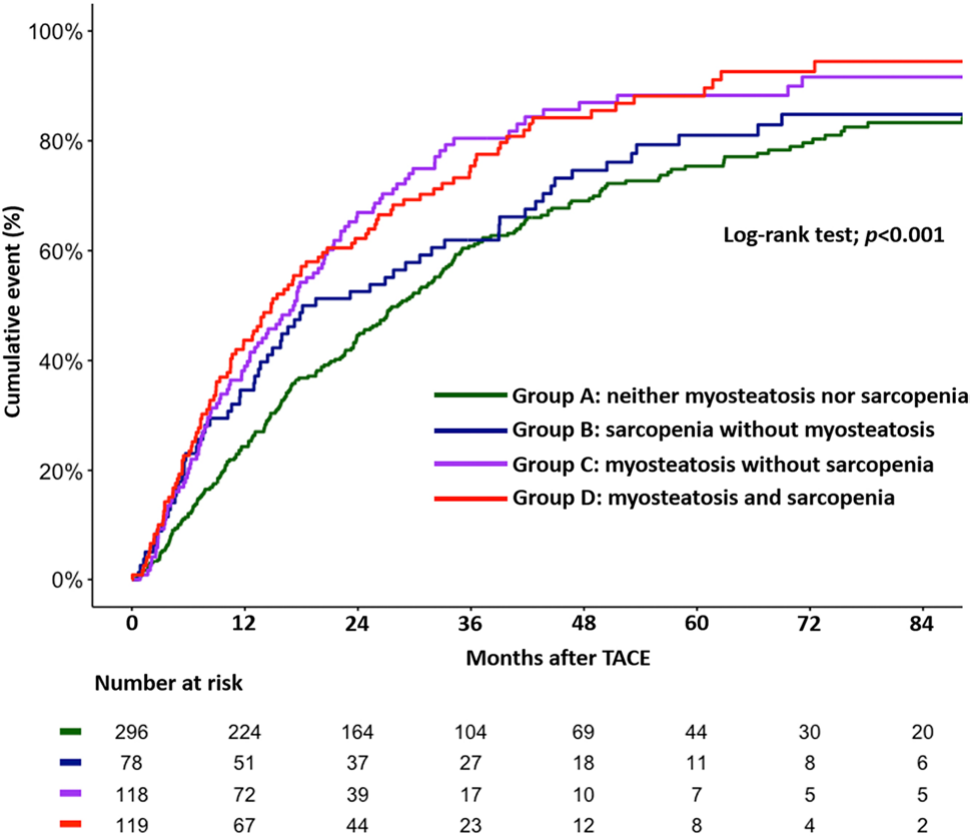
All-cause mortality according to myosteatosis and sarcopenia status.

Patients in group D (myosteatosis and sarcopenia) had the highest mortality rate at seven years (94.45%), whereas group A (neither myosteatosis nor sarcopenia) had the lowest mortality rate (83.31%) (Fig. 4). The mortality rate was 84.82% in group B (sarcopenia without myosteatosis) while it was 91.62% in group C (myosteatosis without sarcopenia). The all-cause mortality rate was significantly different among the four groups (*P* < 0.0001). Compared with group A, the adjusted risk for all-cause mortality was significantly higher in group C (adjusted HR 1.70, 95% CI 1.33–2.17) and group D (adjusted HR 1.75, 95% CI 1.36–2.23). The risk of all-cause mortality in group B was similar to group A (adjusted HR 1.19, 95% CI 0.90–1.57).

## Discussion

We evaluated the potential impact of myosteatosis on outcome in patients with HCC who received TACE as their initial treatment. Our cohort revealed that myosteatosis, as assessed by SMD, was associated with a poor response to TACE and lower survival rates in patients with HCC. The early identification of myosteatosis and the implementation of early preventive strategies may potentially improve the prognosis of patients with HCC.

Several previous studies have demonstrated that sarcopenia and myosteatosis were negative prognostic factors for oncological patients^6,24–26^. HCC is normally concomitant with chronic liver disease and cirrhosis, which have a robust relationship with alterations of body composition^27^. Moreover, most patients with HCC are frequently unfit for curative surgical treatment due to underlying cirrhosis and comorbidities; therefore, TACE remains the treatment of choice in the management of these patients^28^. Although previous cohorts demonstrated that frailty, malnutrition, and loss of muscle mass and function were related to poor results in HCC patients, these patients were usually highly heterogeneous with a variety of available treatments that primarily focused on sarcopenia^11–13^.

In our cohort, we demonstrated that myosteatosis is superior to sarcopenia in being associated with survival outcomes in patients with HCC who underwent TACE. Myosteatosis, rather than sarcopenia, was also recognized as an independent factor associated with mortality after adjusting for clinically significant covariates. The results of our study were similar to previous studies that reported that SMD was a better prognostic factor than SMI in terms of statistical significance in renal, pancreatic, gastric, and breast cancers^6,8,29,30^, which indicated that myosteatosis is a more reliable indicator of association with survival outcomes compared to sarcopenia status. In general, CT-based results permit for early recognition of decreases in SMD even when the SMI has not changed^29^. Therefore, SMD deterioration is identified earlier than a change in SMI.

Sarcopenia is a progressive loss of skeletal muscle mass and strength. Myosteatosis is characterized by the fatty infiltration of muscle tissue that can be identified as low muscle density on CT images and is a contributing component to sarcopenia^31^. SMD and SMI were positively correlated in our cohort, which was consistent with the results of some previous studies^8,32,33^, but contrasted with other studies^34,35^. However, there were some differences in terms of the cut-off values, type of cancer, and stage of disease. There is no consensus on a cutoff value for body composition that is applicable in Asian countries. Thus, we used the cut-off values for SMD and SMI from a study in Japan by Fujiwara et al.^11^, not from a Western study^24^. Furthermore, our study focused particularly on HCC patients in the intermediate stage with a relatively poor prognosis. We found that 19.5% of HCC patients who underwent TACE exhibited a concomitant presence of myosteatosis and sarcopenia with the worst overall survival compared with the other groups (14.8 vs. 23.6 months, *P* < 0.001).

In the present study, myosteatosis was significantly associated with TACE response (*P* = 0.002); however, sarcopenia failed to stratify our patients based on tumor responsiveness. It has been theorized that adipocytes (intermuscular fat) in myosteatosis release inflammatory adipokines that cause impaired nutritive blood flow to muscle, which worsens insulin diffusion capacity and contributes to insulin resistance^36^. As a result, myosteatosis may reduce body immunities, stimulate cancer growth, and affect unfavorable treatment outcomes^37^.

Myosteatosis was shown in this study to be associated with worse overall survival in patients with HCC who underwent chemoembolization. Therefore, it is important to identify patients with higher skeletal muscle fat accumulation before treatment in order to implement early preventive strategies that aim to maintain muscle quality and improve prognosis. Currently, there is no established treatment strategy specifically for myosteatosis in cancer patients; however, some studies have suggested that intensifying perioperative and postoperative exercise, including resistance training, may help maintain postoperative physical strength and lead to earlier resumption of daily activities^38,39^. Additionally, nutritional support that includes vitamin D, omega-3 fatty acids, and β-hydroxy β-methyl butyrate may also help improve muscle mass and quality in cancer patients^40^. Nevertheless, it is important to note that more research is needed to fully understand the relationship between myosteatosis and cancer survival, as well as to determine the optimal treatment strategies for patients with this condition.

This study has the following limitations. First, this study was a single-center retrospective design. While most of the patients in our study received a CT scan before surgery, only one-third of patients had their body composition evaluated one month after TACE, which possibly led to variations in the imaging data. Second, this study was conducted at a tertiary referral center for cancer patients in southern Thailand; therefore, survival rates may vary between specialized and non-specialized centers as well as between different countries and healthcare systems. Further studies should be conducted in other populations to validate the findings of the current study. Finally, the lack of consensus definitions for myosteatosis and sarcopenia is a significant limitation in the field of body composition research in cancer patients. Further research is necessary to establish the optimal threshold for these definitions. This study had a few strengths. Our dataset had a relatively large number of patients and a homogeneous type of treatment modality, which was conventional TACE. Moreover, we not only determined the association of body composition with survival outcome but also established response to TACE in patients with HCC as useful clinical information to aid treatment decisions. Moreover, appropriate perioperative management strategies, such as preoperative respiratory exercise, protein supplementation, and other techniques that enhance skeletal muscle health, can be implemented to lower mortality rates in patients with HCC.

## Conclusion

The presence of myosteatosis was significantly associated with poor TACE response and reduced survival. Identifying patients with myosteatosis and implementing early preventive strategies may preserve muscle quality and potentially improve the prognosis of patients with HCC.

## Data Availability

All data were stored separately in a data repository and are available from the corresponding author on reasonable request.
